# Designing a Polymer-Based Hybrid with Simultaneously Improved Mechanical and Damping Properties via a Multilayer Structure Construction: Structure Evolution and a Damping Mechanism

**DOI:** 10.3390/polym12020446

**Published:** 2020-02-14

**Authors:** Kangming Xu, Qiaoman Hu, Hong Wu, Shaoyun Guo, Fengshun Zhang

**Affiliations:** 1 College of Materials Science and Engineering, Chongqing University of Arts and Sciences, Yongchuan, Chongqing 402160, China; xukangmingaaa@hotmail.com (K.X.); qmhuwit@163.com (Q.H.); 2 State Key Laboratory of Polymer Materials Engineering, Polymer Research Institute of Sichuan University, Chengdu 610065, China; 3 Institute of Chemical Materials, China Academy of Engineering Physics, Mianyang 621900, China; zfs8505@163.com

**Keywords:** hindered phenol, mechanical property, damping property, multilayer hybrids, structure evolution

## Abstract

Though hindered phenol/polymer-based hybrid damping materials, with an excellent loss factor, attract more and more attention, the significantly decreased mechanical property and the narrow damping temperature range limit the application of such promising materials. To solve the problems, a polyurethane (hindered phenol)/polyvinyl acetate multilayer system with varied layer numbers was prepared in this study. The multilayer microstructures were first verified through the scanning electron microscopy. A subsequent molecular dynamics simulation revealed the promoted diffusion of polyurethane (hindered phenol) and polyvinyl acetate layers, the compact chain packing of the polyurethane (hindered phenol) layer, the extended chain packing of the polyvinyl acetate layer, the intermolecular hydrogen bonds among the three components and the enhanced interface interactions between the two layers in a quantitative manner. Further the mechanical and dynamic mechanical analysis detected the successful preparation of the multilayer hybrids with simultaneously improved mechanical and damping properties. Then, by a combination of molecular dynamics simulation and experiment, the relationship between the structure evolution and the properties of the multilayer hybrids was established, which was expected to have some guiding significance for industrial production.

## 1. Introduction

Since it was proposed in 1920 [1], hydrogen bonds (HBs) have attracted enormous attention for nearly a century [2,3,4,5]. The importance of HBs cannot be overemphasized, just as the description given in the Penguin dictionary: “Life would be impossible without this type of bond” [6]. It is responsible for the structure and property of the essential life compound: water, and is the fundamental component of the structure and function of biomolecules, such as proteins, polysaccharides and nucleic acids [7].

In polymer science, HBs also play an important role. Lots of researches have shown that the compatibility [8,9,10], the thermal property [11,12] and the mechanical property [13] of polymer blends are greatly affected with the variation of HBs. Moreover, the significant influence of HBs on the damping property of polymer/hindered phenol (HP) hybrids has also attracted extensive attention in the recent few years [14,15]. For polymer/HP hybrids, reversible HBs form between polymers and HPs. Except the internal friction between molecular chains, the destruction and re-construction of the intermolecular HBs bring additional external energy dissipation during dynamic processes. Thus, the loss factor (tan δ) of the hybrids is significantly improved. Experimental analysis shows that the more intermolecular the HBs are, the larger the tan δ value of the hybrids. For example, Cao et al. found that compared with aggregate HPs, the tan δ value of homogeneous dispersed HPs in hydrogenated butadiene-acrylonitrile rubber was much larger due to the much more intermolecular HBs [16,17]. Zhao et al. then found that because of the large surface area of nanoscale HP aggregates in nitrile rubber, the tan δ of the hybrids with abundant intermolecular HBs also exhibited greatly improved values [18]. Furthermore, to explore the damping mechanism of the nitrile rubber/HP hybrids in a quantitative manner, molecular dynamics (MD) simulation was adopted [19]. The simulation results showed that the largest improvement of the tan δ value was attributed to the largest number of intermolecular HBs and the biggest value of binding energy as well as the smallest fractional free volume. Subsequently, similar simulation results were obtained in studies of other polymer/HP hybrids [20,21,22,23]. Therefore, by regulating the above three factors, a polymer/HP hybrid with an excellent tan δ value and potential application value in the industry can be obtained. However, because of the existence of the apparent defects, especially the significantly decreased mechanical property and the narrow damping temperature range [24,25,26,27,28], the application of such a promising damping hybrid is still limited so far.

As a small molecule with short chains, HPs act as a plasticizer or anti-plasticizer in the polymer matrix, which results in the sharp decrease of tensile strength of the hybrids. Moreover, the relaxation of HP chains is very fast, which is not on the same time scale with that of polymer chains. Thus, the damping temperature range is dominated only by the relaxation of polymer chains and results in a narrow value. To overcome the above defects, one of the effective approaches is the import of a second polymer with appropriate tensile strength and glass transition temperature (*T*_g_) into the binary hybrids. Based on numerous import methods reported before, the construction of the interpenetrating polymer network (IPN) structure seems to be the best way. A system with both a high tan δ value and wide damping temperature range can be obtained by adopting this method [29]. However, the mechanical property cannot be controllable regulated. Besides, the preparation process of the IPN structure is complex. Fortunately, these problems may be solved by a novel multilayered co-extrusion system designed by our lab [30,31,32,33]. The detailed schematic of this equipment is shown in the following experimental section. Through multilayered co-extrusion, a multilayered system, bearing similarity to the IPN structure, can be continuously processed. Moreover, the mechanical property of the shell-like structure may also be controllable regulated [34], the key lies in the strength of the interface interactions, which may be regulated by HBs.

Therefore, to prepare a useful polymer-based hybrid with excellent comprehensive performance, a multilayer system with thermoplastic polyurethane (TPU) and polyvinyl acetate (PVAc) as a matrix was prepared in this study by combining our previous works [35,36]. In order to regulate interface interactions between TPU and PVAc, HP as an HB donor was added into the TPU matrix. Moreover, to explore the damping mechanism with layer evolution at the molecular level, MD simulation was adopted. By a combination of experiment and MD simulation, the purpose of this study is to: (1) prepare a hybrid with excellent properties by combining the advantages of polymer/HP system and multilayered co-extrusion system and (2) reveal the damping mechanism of the multilayered structure at the molecular level and in a quantitative manner.

## 2. Experimental Section

### 2.1. Materials

Polyester-based TPU (Elastollan S70A11), with a hardness of 70 shore A, density of 1.120 g cm^−3^ and 4,4’-methane diisocyanate within the hard segments, was purchased from Elastogran (Lemförde, Germany), BASF group (Lemförde, Germany). PVAc (grade G30) with a molecular weight of 4 × 10^4^–6 × 10^4^ g mol^−1^ and a solid content of 100% was purchased from Wuxi Sincere Chemicals Co., Ltd. (Wuxi, China). Triethylene glycol bis(3-tert-butyl-4-hydroxy-5-methylphenyl)propionate (AO-70), HP in powder form, was obtained from Beijing Additive Research Institute (Beijing, China). All the materials were used without further purification.

### 2.2. Sample Preparation

**Figure 1:**
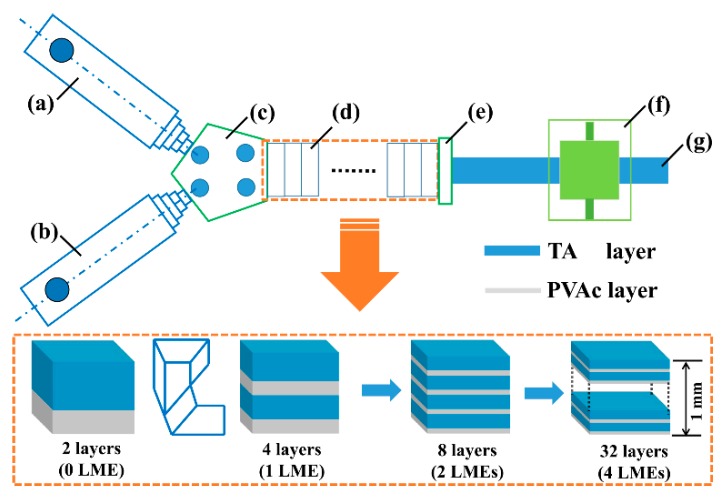
Schematic of multilayered co-extrusion system: (**a**,**b**) single screw extruder; (**c**) co-extrusion block; (**d**) laminating-multiplying elements (LMEs); (**e**) exit block; (**f**) rolling and cooling block and (**g**) multilayer sheet.

The TPU(AO-70)-PVAc multilayer hybrids were prepared as follows: (1) the as-received TPU and AO-70 were dried in a vacuum oven at 80 and 50 °C for 12 and 24 h, respectively and (2) the dried TPU and AO-70 were extruded through a twin-screw extruder (SHJ-20, Nanjing GIANT Machinery Co., Ltd., Nanjing, China) with an extrusion speed of 130 rpm, a temperature gradient of 150–195 °C along the extrusion direction and a mass ratio of 100/25. The extrudate was designated as TA; (3) the TA and the as-received PVAc were dried in an oven at 80 °C and a vacuum oven at 50 °C for 24 h, respectively and (4) the dried TA and PVAc were coextruded through the multilayered co-extrusion system. The schematic of the multilayered co-extrusion system is shown in Figure 1. The TA and PVAc were first simultaneously extruded through different single screw extruders, and then combined as a 2-layer melt in the co-extrusion block. Subsequently, the melt flowed through a series of laminating-multiplying elements (LMEs). The flowing behavior in an LME can be divided into three processes: dividing, stretching and multiplying. At the entrance of an LME, the melt is evenly sliced by a divider into left and right sections, which then enter into two fish-tail channels, respectively. When flowing through a fish-tail channel, the divided section becomes thin and broad. At the exit of the LME, a multiplying process takes place to combine the two separated sections together. An assembly of n LMEs can produce a laminar composite with 2^(n+1)^ layers. After cooling, a multilayer sheet about 1 mm thick, 10 mm wide was produced. In this work, the multilayer hybrids with a layer number of 2, 8, 16 and 32 were produced, which were designated as TP2 to TP32 (TP system), respectively. The mass ratio of TA and PVAc were fixed by fixing the extrusion speed. The temperatures of extruders for TA and PVAc were 195 and 140 °C, respectively. The temperature of LMEs was 190 °C; (5) in order to eliminate the potential chain orientation, the multilayer hybrids were treated by annealing at 150 °C for 10 min [37]. Besides, a pure TPU-PVAc 8-layer system, named PTP8, was also prepared along with the above preparation method for comparison. 

### 2.3. Characterization

#### 2.3.1. Morphological Observation

A scanning electron microscopy (SEM, JSM-5900LV, Tokio, Tokyo, Japan) was used to observe the morphology of the multilayer hybrids. All samples were first polished through a freezing microtome (RM2265, Leica, Weztlar, Germany) along the direction perpendicular to the extrusion, then before testing, the polished surface was sputter-coated with Au.

#### 2.3.2. Differential Scanning Calorimetry (DSC)

The thermal properties of the multilayer hybrids (5–7 mg) were measured by means of differential scanning calorimetry (DSC; Q20, TA Instrument, Newcastle, DE, USA). Nitrogen gas was purged while the measurements were being taken. The sample was first heated from room temperature to 210 °C to eliminate the heat history. Subsequently, the sample was cooled to −80 °C and heated again to 210 °C (second heating). All the heating and cooling rates were 10 °C min^−1^. The glass transition temperature (*T*_g_) was obtained from the second heating scan.

#### 2.3.3. Mechanical Property Testing

Tensile properties of the multilayer hybrids were tested using a universal material experiment machine (CMT-4104, MTS, Eden Prairie, MN, USA) with a rate of 50 mm/min and a temperature of 25 ± 1 °C in accordance with ASTM D638. Five specimens for each hybrid were tested and the average value was calculated.

#### 2.3.4. Dynamic Mechanical Analysis (DMA)

Dynamic mechanical spectra were acquired using a dynamic mechanical analyzer (Q800, TA Instrument, Newcastle, DE USA). The samples with sizes of 100 mm (length) × 10 mm (width) × 1 mm (thickness) were heated from −60 to 120 °C at a constant frequency of 10 Hz and a heating rate of 3 °C min^−1^ under a double-cantilever mode.

### 2.4. Simulation Strategies

MD simulation was performed for TPU, PVAc, AO-70, TA and the multilayer composites at ambient temperature (25 °C) using the Discover, Forcite and Amorphous cell modules (Accelrys, Inc., San Diego, CA, USA) of the Material Studio Modeling software (version 7.0, Accelrys, Inc., San Diego, CA, USA). The condensed-phase optimized molecular potentials for an atomistic simulation studies (COMPASS) force field was used for computing the interatomic interactions [38]. The temperature and pressure regulation depended on the Andersen and Berendsen method [39]. The non-bonded interaction and van der Waals interactions were determined by the standard atom-based simulation method [40]. Taking into account the structure of the practice polymer used in the experiment, a predigested method was used to construct the TPU structure in this paper [35]. TPU and PVAc oligomers consisting of 6 and 20 repeating units were adopted in this simulation [41].

**Figure 2:**
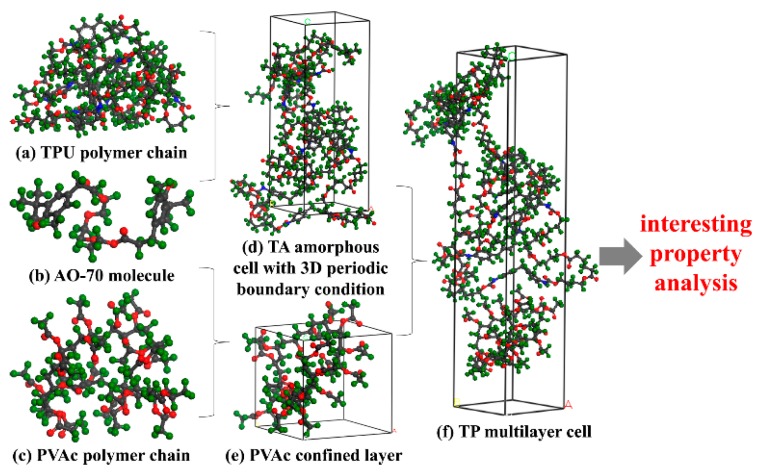
Models for molecular dynamics (MD) simulation of the multilayer composites (red atom is O, green atom is H, grey atom is C and blue atom is N). (**a**) TPU polymer chain; (**b**) AO-70 molecule; (**c**) PVAc polymer chain; (**d**) TA amorphous cell with 3D periodic boundary condition; (**e**) PVAc confined layer; (**f**) TP multilayer cell.

Figure 2 shows the process of MD simulation. Initially, the proposed structure of TPU, PVAc and AO-70 were generated by using the rotational isomeric state model of Flory, then initial structures were ordinal energy minimized through the steepest descent, conjugate gradient and newton method to a convergence level of 1 × 10^−5^ kcal/mol/A with maximum iterations of 4 million, and then the structures were dynamic equilibrated in a constant NVT (Constant number, Constant volume, constant temperature) condition at 25 °C for 5 nanosecond (ns) with a time step of 1 femtosecond (fs; Figure S1, from the Supplementary Materials) to avoid excessive overlaps between chains (Figure 2a–c). Subsequently, the TA amorphous cells and PVAc confined layers with a C (or Z) cell dimension ratio similar to the experiment were constructed, respectively. Moreover, in order to pile the following layer cells correctly, the amorphous cells and confined layers were selected to have almost the same A (or X) and B (or Y) cell dimensions. Then the amorphous cells and confined layers were ordinal energy minimized through the steepest descent, conjugate gradient and newton method to a convergence level of 1 × 10^−5^ kcal/mol/A with maximum iterations of 4 million, and then the amorphous cells and confined layers were dynamic equilibrated in a constant NVT condition at 25 °C for 4 ns with a time step of 1 fs (Figure 2d,e and Figure S2 taking a TA-32 layer and PVAc-32 layer for example, from the Supplementary Materials). After that, the layer cells with PVAc as the bottom wall and TA as the fluid were piled. The layer cells were also ordinal energy minimized through the steepest descent, conjugate gradient and newton method to a convergence level of 1 × 10^−5^ kcal/mol/A with maximum iterations of 4 million, and then the layer cells were dynamic equilibrated in a constant NVT condition at 25 °C for 2 and another 4 ns with the trajectories being saved every 5 picoseconds (ps; Figure 2f and Figure S3 taking TP32 for example, from the Supplementary Materials). At last, the last equilibrated 4 ns could be used to calculate the properties of interest.

## 3. Results and Discussion

### 3.1. Microstructure and Compatibility

**Figure 3:**
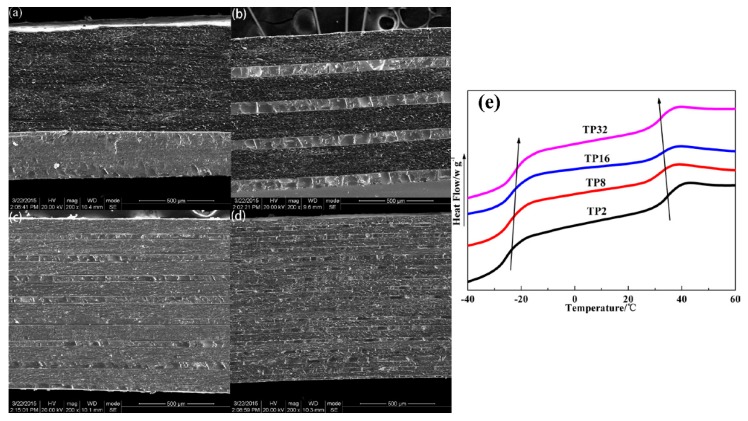
(**a**) SEM image of TP2; (**b**) SEM image of TP8; (**c**) SEM image of TP16; (**d**) SEM image of TP32 and (**e**) differential scanning calorimetry (DSC) curves of TP hybrids.

To verify whether the multilayer structure was successfully constructed and the compatibility variation of TP hybrids with a layer number increasing, SEM and DSC were employed. For SEM results in Figure 3a–d, all TP hybrids exhibit distinct co-continuous layered morphology, where the TA layers are relatively rough because of its inherent flexible features and the PVAc layers are relatively smooth because of its inherent rigid features. Moreover, the layer thickness of TA and PVAc decreases with a layer number increasing at an almost unchanged thickness ratio, indicating the successful construction of the multilayer structure. Besides, the successful construction of the PTP8 multilayer system was also confirmed as Figure S4 shows. For the DSC result in Figure 3e, two transitions corresponding to *T*_g_ of the soft segments of TPU and PVAc were detected for all hybrids. With the layer number increasing, the two transitions shifted to each other, indicating the improvement of the compatibility between the TA and PVAc layer. Moreover, neither the melting peak nor the glass transition at 6.82 °C of AO-70 is observed in the curves [35,36], which indicates that AO-70 is miscible with TPU or PVAc in the form of the amorphous state in all multilayer hybrids.

### 3.2. MD Simulation Analysis

To explore the structure variation of the multilayer hybrids with a layer number increasing at the molecular level for a further mechanism study, MD simulation based on amorphous cells was adopted. The detailed simulation results of possible location of chains, agglomeration degree of chains, HBs variation and adhesion strength of layers are shown below.

#### 3.2.1. Possible Location of Chains

**Figure 4:**
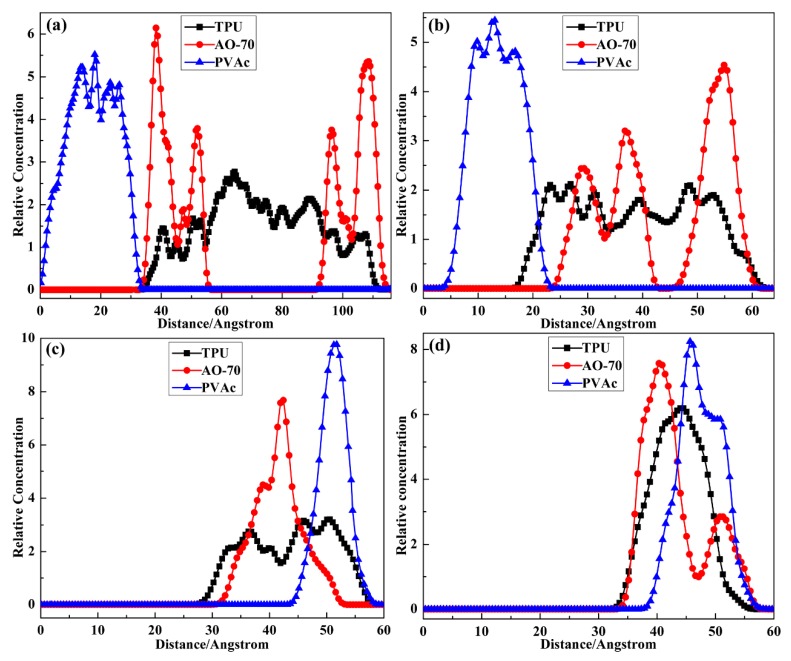
Relative concentration of chains along the c axis of the layer cells: (**a**) TP2; (**b**) TP8; (**c**) TP16 and (**d**) TP32.

To explore the structure evolution with a layer number increasing, especially the interface variation between TA and PVAc layers, the possible location of chains along the thickness direction of the cells was obtained by analysis of the relative concentration along the C (or Z) axis of the layer cells. The results are shown in Figure 4. For TP2 with the largest layer thickness, the relative concentration of TPU and PVAc chains show a clear boundary, indicating the weak diffusion of the two chains at the layer interface. The relative concentration of AO-70 chains was uneven and mainly dispersed in the TA layer, which indicates the weak diffusion and interaction between AO-70 and PVAc chains. For TP8 with the layer thickness decreasing, the relative concentration of TPU and PVAc chains show overlapping, indicating the enhanced diffusion of the two chains at the layer interface. In addition, attributed to the decreased TA layer thickness, the relative concentration of AO-70 chains was uniform, while AO-70 was still mainly dispersed in the TA layer. By the way, the enhanced diffusion of TPU and PVAc chains at the interface also existed in TPT8 though the result was not presented here. For TP16 and TP32 with further layer thickness decreasing, the relative concentration of TPU and PVAc chains show more and more overlapping, indicating the further diffusion of the two chains at the layer interface. Moreover, the relative concentration of AO-70 chains also shows overlap with that of PVAc chains, indicating the improved diffusion of AO-70 chains from the TA to PVAc layer, which therefore may result in the improved interaction between AO-70 and PVAc.

#### 3.2.2. Agglomeration Degree of Chains

**Figure 5:**
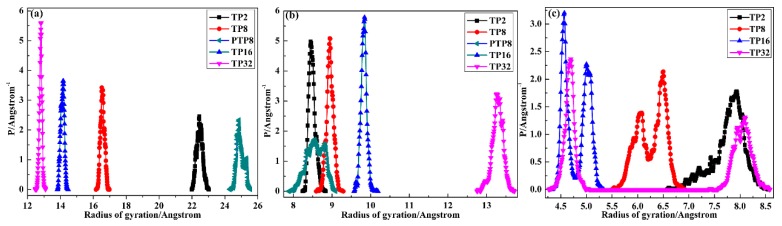
Radius of gyration probability distribution of chains in the layer cell: (**a**) TPU; (**b**) PVAc and (**c**) AO-70.

To explore the agglomeration degree variation of TPU, PVAc and AO-70 chains with a layer number increasing, the radius of gyration (*R*_g_) distribution was examined and the results are depicted in Figure 5. For *R*_g_, a small value represents compact chain packing while a large value represents extended chain packing [42]. The *R*_g_ value of TPU chains for the TP system gradually decreased with layer thickness decreasing, indicating the more and more compact chain packing of TPU, while the *R*_g_ value of PVAc chains for TP system gradually increased, indicating the more and more extended chain packing of PVAc. Moreover, compared with the *R*_g_ value of TP8 and PTP8, the compact chain packing of TPU and the extended chain packing of PVAc were mainly attributed to the introduction of AO-70. The *R*_g_ value of AO-70 chains gradually decreased from TP2 to TP16, while that for TP32 shows an uneven distribution. Combined with the possible location results above, the uneven distribution was mainly attributed to the diffusion of AO-70 chains from the TA to PVAc layer. 

#### 3.2.3. Hydrogen Bond Evolution

**Figure 6:**
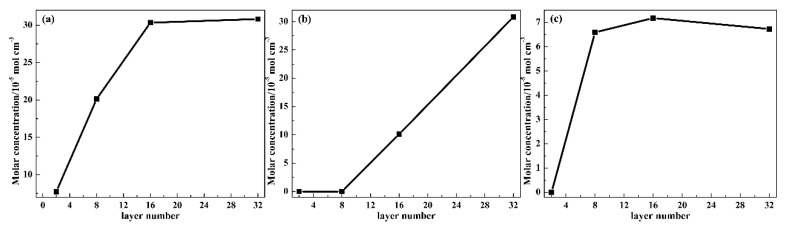
(**a**) Intermolecular *C*_HBs_ between the phenolic O–H group in AO-70 and the ester C=O group in the soft segment of TPU; (**b**) intermolecular *C*_HBs_ between the phenolic O–H group in AO-70 and the ester C=O group in PVAc and (**c**) intermolecular *C*_HBs_ between the urethane N–H group in the hard segment of TPU and the ester C=O group in PVAc of TP system at 298 K.

Related to the chemical structure of TPU, PVAc and AO-70, two types of proton donators (i.e., phenolic O–H group in AO-70 (1) and urethane N–H group in the hard segment of TPU (2)) and four types of proton acceptors (i.e., urethane C=O group in the hard segment of TPU (3), ester C=O group in the soft segment of TPU (4), ester C=O group in PVAc (5) and ester C=O group in AO-70 (6)) exist. The possible intermolecular HBs within 1–3, 1–4, 1–5, 2–5, 2–6 and the intramolecular HBs within 1-6 have great impacts on the properties. Therefore, to explore the above possible HBs, five repeated cells obtained by the repeated MD simulation condition were used to obtain the number of HBs, and then the molar concentration of HBs (*C*_HBs_) of TP system was obtained. The results are shown in Figure 6. *C*_HBs_ is calculated using the following equation:

(1) CHBs=NHBsNAV

where *N*_HBs_, *NA* and *V* are the statistical average number of HBs in the periodic cell, Avogadro’s constant and the volume of the periodic cell, respectively. With the layer number increasing, the *C*_HBs_ between 1 and 4 sharply increased from TP2 to TP16 and then reached a maximum value at TP32, which resulted in the more and more compact chain packing and indicates the enhanced intermolecular HBs between AO-70 and TPU. The *C*_HBs_ between 1 and 5 shows a gradual increase at TP16 and TP32 from zero, which was mainly attributed to the improved diffusion of AO-70 chains from the TA to PVAc layer. The *C*_HBs_ between 2 and 5 fluctuated in a small range except for TP2, indicating the intermolecular HBs between TPU and PVAc changed a little with the layer thickness decreasing. Besides, the intermolecular HBs within 1–3, 2–6 and the intramolecular HBs within 1–6 were not detected for all TP systems. Therefore, the results were not depicted here.

#### 3.2.4. Adhesion Strength of Layers

To explore the interface interaction variation between TA and PVAc layers with the layer number increasing, the overall adhesion energy (*E*_adh_) was calculated. The definition of *E*_adh_ is:

(2) $$E_{\mathrm{adh}}=\frac{E_{\mathrm{inter}}}{A_{\mathrm{vdw}}}$$

where the *E*_inter_ could be defined as:

(3) $$E_{\mathrm{inter}}=E_{\mathrm{total}}—(E_{\mathrm{TA}}+E_{\mathrm{PVAc}})$$

**Figure 7:**
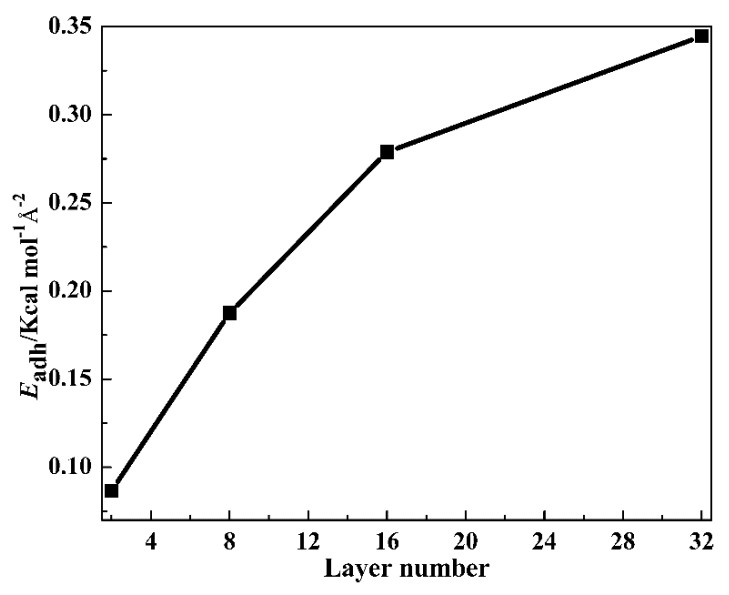
The overall adhesion energy of the TP system.

And the *A*_vdw_ term is calculated via the solvent-excluded surface, or the Connolly surface [43], which is determined by rolling a ball of a particular radius along the surface of the PVAc confined layer. In Formula (3), *E*_inter_ is the overall interaction energy, *E*_total_ is the total potential energy of the layer cell, *E*_TA_ is the internal energy of the TA layer and *E*_PVAc_ is the internal energy of the PVAc layer. Figure 7 presents the *E*_adh_ of the TP system. Attributed to the enhanced chain diffusion and enhanced intermolecular HBs between TA and PVAc layers, the *E*_adh_ gradually increases with the layer thickness decreasing, indicating the improved interface interactions between TA and PVAc layers. In addition, compared with the *E*_adh_ of PTP8 with a value of 0.124 kcal mol^−1^Å^−2^, the *E*_adh_ of TP8 exhibits a much higher value, which is mainly attributed to AO-70.

### 3.3. Mechanical and Dynamic Mechanical Properties

**Figure 8:**
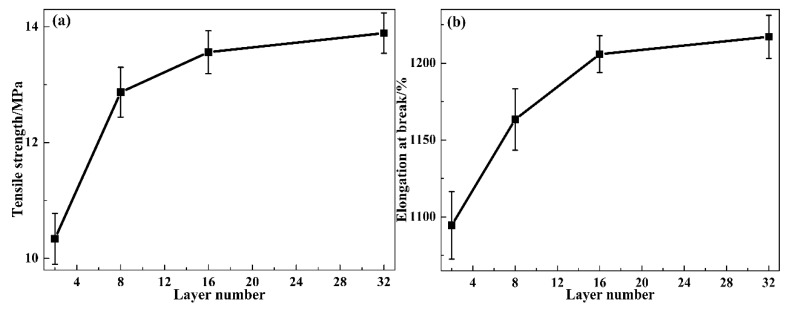
Mechanical properties of the TP multilayer system: (**a**) tensile strength and (**b**) elongation at break.

Based on all the above simulation results, the TA layer, the PVAc layer and the layer interface show significant changes with the layer number increasing. To investigate the effects of the structure variation on mechanical properties, the mechanical property testing was conducted on the universal material experiment machine and the results are shown in Figure 8. With the layer number increasing, the tensile strength firstly shows a dramatic increase from 10.3 for TP0 to 12.9 MPa for TP8, then gradually increased to 13.6 and 13.9 MPa for TP16 and TP32, respectively. The elongation at break also firstly shows a dramatic increase from 1094.6% for TP0 to 1205.8% for TP16, then gradually increased to 1217.2% for TP32. Besides, the tensile strength and elongation at break for PTP8 were 27.0 MPa and 925.1%, respectively. As mentioned in the introduction, HPs act as a plasticizer or anti-plasticizer in HP/polymer binary hybrids, which leads to the dramatic decrease of tensile strength and the dramatic increase of elongation at break of the hybrids [14,15,16,17,18,19,20,21,22,23,24,25,26,27]. Thus, the difference of the tensile strength and elongation at break between TP8 and PTP8 was mainly attributed to the plasticizing effect of AO-70 to TPU. For the TP system with the layer thickness decreasing, the enhanced chain diffusion and intermolecular HBs led to the improved interface interactions between TA and PVAc layers, which resulted in a better force transfer between the two layers. Moreover, the extended chain packing resulted in the improved tensile strength of PVAc and the compact chain packing resulted in the improved elongation at break of TPU. Thus, combined with the variation of the interface interactions, the PVAc layer and TA layer, the tensile strength and elongation at break of the TP system show dramatic increases. In addition, the gradual increase of tensile strength and elongation at break might be attributed to the enhanced plasticizing effect of AO-70 to PVAc and the variation of intermolecular HBs between AO-70 and TPU, respectively.

**Figure 9:**
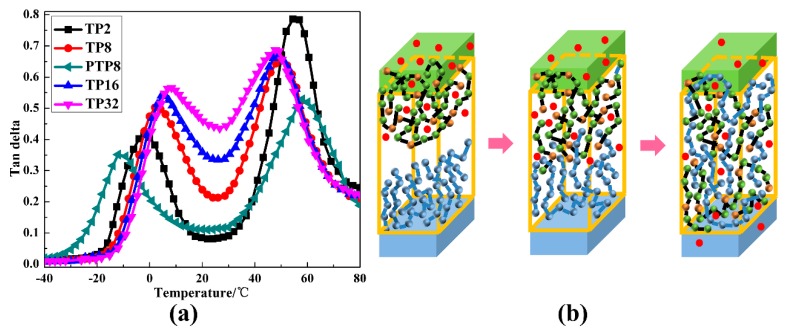
(**a**) The tan δ versus temperature curves of the TP and PTP multilayer system and (**b**) the structural schematic diagram of the TP system (cyan ball and line represent the PVAc chain at interface, cyan layer represents the PVAc layer, red ball represents AO-70, green layer represents the TPU layer and green and yellow ball combined with a black line represent the TPU chain at interface).

To further explore the effects of the structure variation on the damping properties (i.e., tan δ and the temperature range of tan δ > 0.3) and the related damping mechanism, the temperature dependence of tan δ of the TP and PTP system was conducted on the dynamic mechanical analyzer and the results are shown in Figure 9a and Table 1. Besides, the structural schematic diagram of the TP system is depicted in Figure 9b for a better understanding. In Figure 9a, two tan δ peaks corresponding to the *T*_g_ of the soft segments of TPU and PVAc, and one wave trough between the two peaks were detected in all of the hybrids. For the TP system with the layer number increasing, the two peaks shifted to each other, which was consistent with the DSC results. The tan δ_max_ value of TPU gradually increased from 0.42 to 0.56 while that of PVAc firstly decreased from 0.79 to 0.66 then slightly increased to 0.68. More importantly, the wave trough dramatic increased with the layer thickness decreasing, which resulted in the significant improvement of the temperature range of tan δ > 0.3. However, compared with TP8, the tan δ peak of TPU and PVAc for PTP8 was located at a lower and higher temperature, respectively. In addition, the tan δ_max_ value of TPU and PVAc were much lower. Moreover, the wave trough for PTP8 also exhibited a low value, which resulted in a low value of the temperature range of tan δ > 0.3. Obviously, the introduction of AO-70 into the TP system had a positive impact on the damping properties. For the TP system with the layer thickness decreasing, the compact chain packing of TPU led to the enhanced internal friction of chains and the improved intermolecular HBs between TPU and AO-70 also brought additional energy dissipation, thus the tan δ_max_ value of TPU shows a significant increase. The decrease of the tan δ_max_ value of PVAc was mainly attributed to the enhanced effects of TPU chains to PVAc chains by the increase of the layer number. By a comparation of TP8 and PTP8, the effects of TPU chains to PVAc bulk will weaken with the compact chain packing of TPU chains, which results in a better tan δ_max_ value of PVAc. Moreover, the effects are mainly focused on the interface. Due to the enhanced chain diffusion and the enhanced intermolecular HBs between PVAc and AO-70, the interface interactions between TA and PVAc layers greatly improved, which led to the significant improvement of the wave trough value. As a result, a multilayer hybrid with a high damping value and wide damping temperature range was achieved. 

## 4. Conclusions

To conclude, by a combination of MD simulation and experimental analysis, the layer structure evolution of the TP multilayer system along with the layer thickness decreasing and the relationship with (dynamic) mechanical properties were studied in detail for the first time. The decrease of layer thickness led to a promoted diffusion between the TA and PVAc layer, a compact chain packing of the TA layer and an extended chain pack of the PVAc layer, which resulted in the formation of intermolecular HBs networks among AO-70, TPU and PVAc, and an enhanced interface interaction between the TA and PVAc layer. Therefore, the mechanical and dynamic mechanical properties of the multilayer hybrids show an increase. In addition, a multilayer hybrid with an improved mechanical property, high damping value and wide damping temperature range was achieved. Besides, the formation of intermolecular H-bonds between AO-70 and PVAc led to the extended chain packing of AO-70 at layer interface, which resulted in the improved stability of AO-70 in the hybrids.

## Figures and Tables

**Table 1:** The damping properties of the TP and PTP multilayer system.

Sample Code	Tan δ_max_ (TPU)	*T*_g_ (TPU)	Tan δ_max_ (PVAc)	*T*_g_ (PVAc)	Temperature Range of Tan δ > 0.3
TP2	0.42	−2.16	0.79	57.91	42.24
TP8	0.50	2.44	0.66	52.34	55.62
PTP8	0.35	−12.01	0.53	57.98	32.17
TP16	0.54	3.79	0.67	51.22	68.47
TP32	0.56	4.47	0.68	50.18	68.55

## Supplementary Materials

The following are available online at https://www.mdpi.com/2073-4360/12/2/446/s1, Figure S1: Dynamic equilibrated energy variation vs. simulation time: (1) TPU chain; (2) PVAc chain and (3) AO-70 chain. Figure S2: Dynamic equilibrated energy variation vs. simulation time: (1) TA-32 amorphous cell and (2) PVAc-32 confined layer. Figure S3: Dynamic equilibrated energy variation vs. simulation time of TP32: (1) the first 2 ns and (2) the last 4 ns. Figure S4: SEM image of PTP8.
