# Supplementary material for: Designing a Polymer-Based Hybrid with Simultaneously Improved Mechanical and Damping Properties via a Multilayer Structure Construction: Structure Evolution and a Damping Mechanism
Source: Polymers (Basel). 2020 Feb 14;12(2):446. doi: 10.3390/polym12020446 (PMC7077642; doi:10.3390/polym12020446)
Supplement: Supplementary file 1 [file polymers-12-00446-s001.pdf]

Supplementary Materials:

Designing A Polymer-Based Hybrid with Simultaneously Improved Mechanical and Damping Properties via Multilayer Structure Construction: Structure Evolution and Damping Mechanism

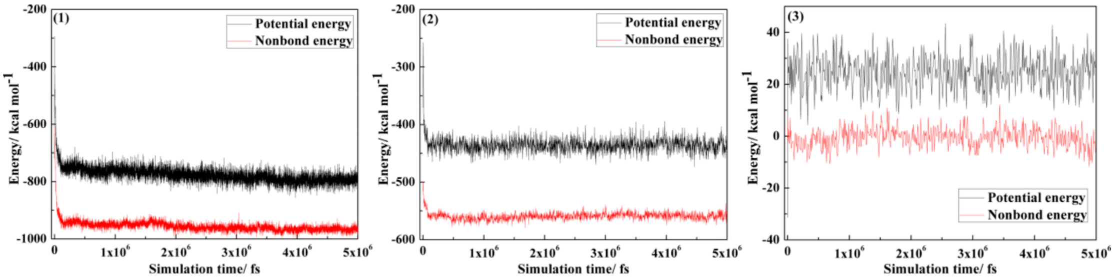

Figure S1. Dynamic equilibrated energy variation vs. simulation time: (**1**) TPU chain; (**2**) PVAc chain and (**3**) AO-70 chain.

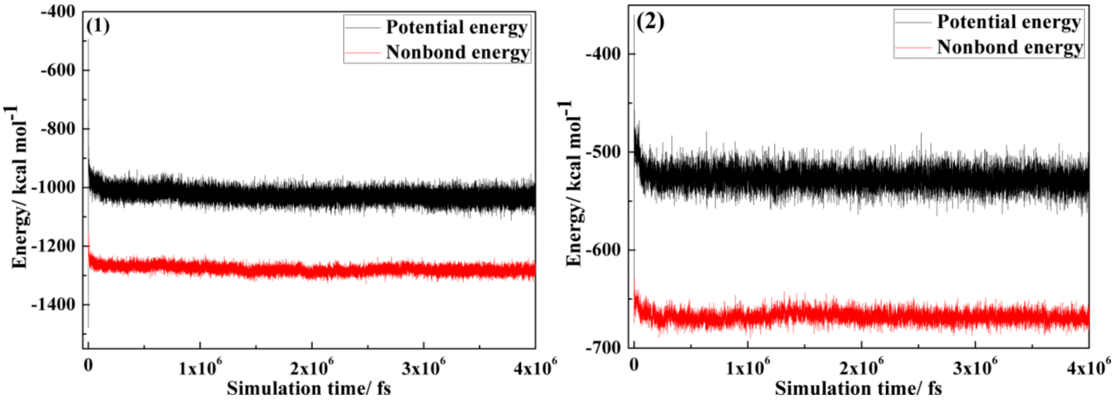

Figure S2. Dynamic equilibrated energy variation vs. simulation time: (**1**) TA-32 amorphous cell and (**2**) PVAc-32 confined layer.

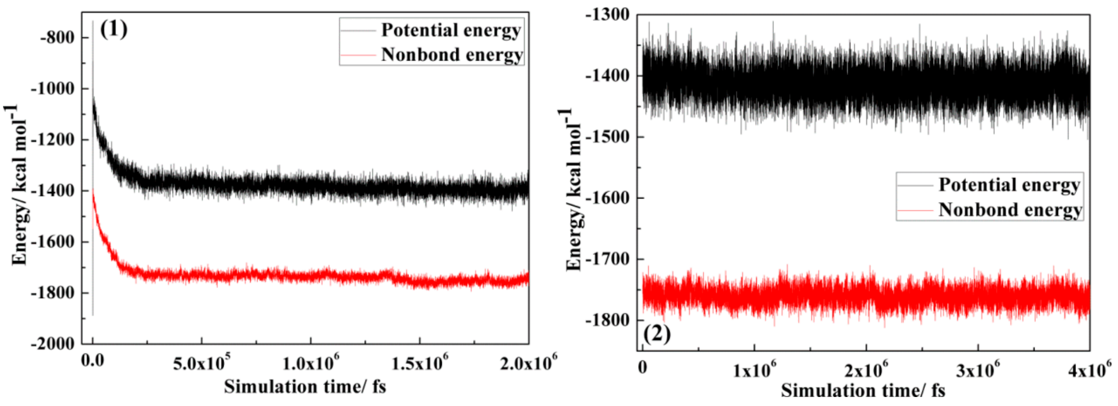

Figure S3. Dynamic equilibrated energy variation vs. simulation time of TP32: (**1**) the first 2 ns and (**2**) the last 4 ns.

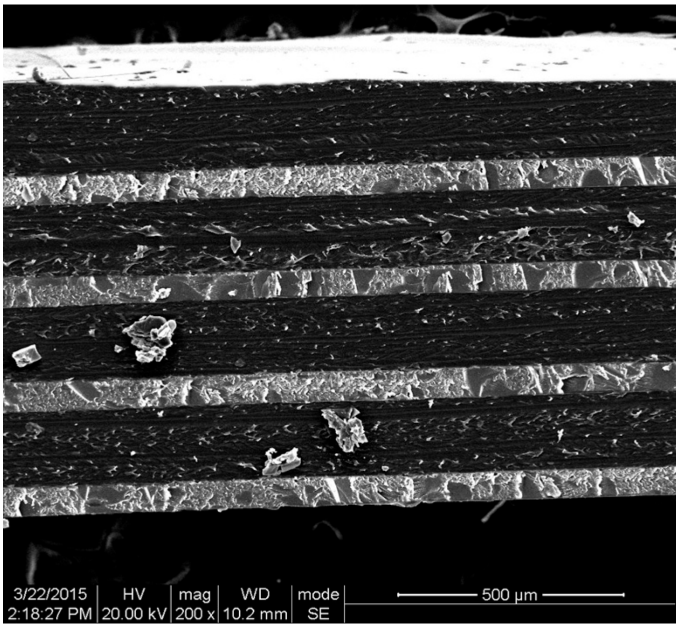

Figure S4. SEM image of PTP8.
